# The role of open data in digital society: The analysis of scientific trending topics through a bibliometric approach

**DOI:** 10.3389/fsoc.2023.1134518

**Published:** 2023-02-01

**Authors:** Maria Carmela Catone

**Affiliations:** Department of Sociology, University of Barcelona, Barcelona, Spain

**Keywords:** open data, bibliometric analysis, research trends, conceptual structure, science mapping

## Abstract

The analysis of contemporary society, characterized by technological, economic, political, social, and cultural changes, has become more challenging due to the development of the internet and information and communication technologies, which provide a vast and increasingly valuable source of information, knowledge, and data. Within this context, so-called open data—that is, data that are made public, especially by public administrations, through an open governance model (transparent and accessible to citizens) are assuming a significant role. This is a topic of growing importance that scientific research is addressing in an attempt to discern the multiplicity of social, educational, legal, technological, statistical, and methodological issues that underlie the creation and use of such data. This article aims to provide insights into understanding scientific trends on the topic of open data through a bibliometric approach. Specifically, a total of 3,110 publications related to the disciplinary fields of the social sciences and humanities published from 2013 to 2022 were collected. The data was then analyzed using network and factorial analysis techniques to detect the conceptual structure to identify the trends of topics and perspectives of research that characterize open data studies.

## 1. Open data landscape: Perspectives and contexts of application

The development of the internet and new technologies has encouraged the growth of mechanisms for producing digital traces and data and has influenced the way this information is managed and shared within public institutions and administrations, as well as in private enterprises. In recent years, a number of changes have taken place in the transparency processes of public administrations, government bodies, and organizations that are now making some data that were previously intended to be used exclusively for administrative procedures, public, free, and reusable. This phenomenon, known as open data, thus refers to data collected within the context of the actions of public administrations that are subsequently made available, reusable, and openly accessible to the community, allowing citizens and stakeholders to gain direct insight into the knowledge of certain public affairs. It is based on a model of “open” and collaborative administration, in which citizens can actively participate in decision-making and knowledge co-creation processes, fostering civic sense, the principles of active and aware citizenship, legality, territorial identity and collaboration.

Open data, intended as the result of a multiplicity of processes involving, on the one hand, increasing technological advances and on the other, the development of the knowledge society, cover a wide range of sectors, from environmental data to demographic statistics, employment, education, etc., and have specific formats and characteristics. The collaborative vision underlying the culture of open data is also supported and enabled by national and international initiatives, such as the latest Directive 2019/1024 of the European Parliament, which aims to improve access to public sector data in order to stimulate their use, both for commercial and non-commercial purposes. The European Data Portal, where metadata of public data made available across Europe are systematically collected to improve accessibility and increase the value of open data, is also exemplary of this approach.

The use of open data plays an important role in the understanding and management of some societal phenomena; for example, its use was particularly evident during the COVID-19 pandemic (Alamo et al., 2020). Throughout the complex and unprecedented health emergency, there was a continuous process of collecting, correcting, integrating, validating, and sharing data that proved useful to the world of journalism and directly to citizens themselves; it enabled government and local administrations to monitor the progress of the pandemic by promoting forecasting and analysis activities.

Clearly, the issues related to the processes of open data creation, dissemination, and analysis are varied and span with technological, regulatory, ethical, economic, educational, cultural, social, and governance aspects that are being studied and applied by the various actors of scientific research. The latter are in fact particularly interested in this phenomenon, since a significant proportion of their activities is based on the collection, processing, and examination of information, which then triggers scientific innovation processes (Berghmans et al., 2017; Fischer et al., 2022). In this perspective, such data activism is supported not only by the trend of open governance but also by scientific research moving toward open science, characterized by processes of accessibility of public scientific knowledge and new forms of democratization of knowledge (Raffaghelli, 2017). At the same time, the development of digital technologies has favored further quantification processes to encourage evidence-based science in the formulation and definition of policies or to extend the logic of the market in the governance of society (Amaturo and Aragona, 2019, p. 72).

Focusing on one of the main characteristics of open data, namely data sharing, it can be stated that it is not a new phenomenon within scientific communities; it represents a founding element of scientificity processes in terms of the publicity of the procedures adopted, accountability on the part of the researcher, and the possibility of generating new results (Gurstein, 2011; Hossain et al., 2016). For example, in scientific research, the interoperability and networked openness of research data are the subject of debate since access to data enables the creation of new datasets by combining data from different sources and encourages diversity of analysis and opinion (Cassella, 2013).

According to many studies (Zuiderwijk et al., 2014; Gonzalez-Zapata and Heeks, 2015; Hossain et al., 2016; Burgelman et al., 2019), open data can be examined from different research perspectives: using the classification adopted by Zuiderwijk et al. (2014), a technical approach explores the infrastructural characteristics of the technologies used as well as aspects related to standardization, interoperability, and data accessibility (Hossain et al., 2016) (e.g., the problems associated with managing open data, based on multiple servers; the procedures for extracting and converting this data into “visualizations” that are understandable to non-expert users, etc.). In a broader sense, it is also concerned with issues related to the digital divide that affect the processes of data creation and use. A different approach to open data is through the social perspective, which examines the impact of open data on public services through activities of data monitoring and transparency carried out by different actors. This research perspective concerns the roles of citizens who become participants in public affairs decision-making processes, promoting principles of active citizenship and civic empowerment. At the same time, as Raffaghelli (2017, p. 301) reminds us, some studies also investigate how such forms of participation and active citizenship could be the basis for forms of public ownership and resistance to power (Baack, 2015). In addition, the social perspective is closely related to the educational approach, which concerns the role played by “data literacy” skills, i.e., the ability to use, interpret, and communicate quantitative information that fosters informed knowledge in the diverse contexts of the increasingly connected and complex contemporary world. With respect to the latter two perspectives, various initiatives toward open data activism and education come to mind, such as Hackathons, and e-learning programs developed by the European Data Portal and other activities promoted by the European Community such as The Data Europe Academy aimed at familiarizing citizens with open government policies and the participatory and accountable science approach (Attard et al., 2015; Raffaghelli, 2017).

Following the classification proposed by Zuiderwijk et al. (2014), another fundamental perspective is the political approach, connected to the analysis of concepts of transparency and “accountability” that institutions also intend to convey, for instance, to restore and strengthen trust between institutions and citizens. Moreover, as indicated by Corazza (2020), some studies (Lupton, 2014, p. 109) deal with the spread of open data which also underlies the neoliberal assumptions that characterize some aspects of the discourse on technologies. Closely intertwined with political studies is the strictly regulatory-legal research approach aimed at understanding policies, regulatory frameworks, directives, and laws ranging from digital rights, for example, to privacy of information and copyright, up to the multiple issues for the creation of the unique global market for data exchange and re-use. One further viewpoint can be added to these, the economic perspective (Huyer and van Knippenberg, 2020), which analyses the impact of open data with respect to macro and micro economic effects (e.g., the personalisation and diversification of services and the reduction of barriers to entry into the markets), possible benefits (e.g., the generation of new products and services and process improvement), and limitations (e.g., related to open data cost-ownership) (Huyer and van Knippenberg, 2020).

Within this broader scenario, the aim of this paper is to explore the main scientific trends and uses of open data by investigating the scientific literature in the field of social and human sciences produced between 2013 and 2022 on this phenomenon. The idea of this article is to understand how the phenomenon of open data, which connects the growing process of digitisation with the production of data, is explored, studied and utilized in disciplinary areas other than the “hard” sciences, that is in the fields of the social sciences and humanities which in recent times are increasingly approaching the culture of data, due to the multiplicity of implications (ethical, social, political, etc.) both at a micro and macro level, that derive from it. Through an exploratory and holistic overview based on bibliometric analysis, the trends of topics and perspectives of research characterizing scientific production relating to open data in the last 10 years are examined. In particular, the evolution of research topics over time and the conceptual structures underlying the study of “open data”—that is, the main themes, subthemes, and patterns of the area—are identified.

These aspects are illustrated in the following paragraphs. More specifically, the next section deals with the methodology of this research, focusing on the data collection and analysis techniques carried out using the bibliometrics approach. Section 3 presents the main findings, exploring in particular the conceptual structure underpinning the domain of “open data.” The last section contains a final discussion and concluding remarks.

## 2. Methodology

In this work, a bibliometric analysis was carried out to explore the scientific debate and the main research trends in the disciplinary fields of the social sciences and humanities on the topic of open data. This approach deals with the measurement and analysis of science and it takes account of the studies of Derek J. de Solla Price and Eugene Garfield who founded the Institute for Scientific Information in the 1960s. These analyses allow us to identify the internal structure and dynamic aspects of scientific research in a specific domain and to map the multiple dimensions and perspectives that characterize a discipline. In particular, the conceptualization of science through a bibliometric approach is based on the idea of a multidimensional construct in which patterns and trends dynamically converge, and it is particularly useful for observing and understanding the multidisciplinary characteristics of a subject domain. In addition, the structures of the scientific literature and the underlying specialities are detected analytically, also using graphical representations that allow “visualization of the topology of the relationships between elements or aspects of science” (Rip, 1988, p.254).

For this research, 3,110 publications produced from 2013 to 2022 were analyzed using a bibliometric approach. These publications were extracted from the Web of Science (WoS) by identifying when the topic “open data” or “open government data” was present in the title, abstract, or used as keywords. Moreover, the documents were selected from the WoS categories of the social and human sciences disciplines, were co-authored by 10,239 different scholars and had 8,139 author's keywords. The data were analyzed using Bibliometrix, an open-source tool for quantitative research in bibliometrics written in the R language and developed by Aria and Cuccurullo (2017).

The analysis was first carried out using univariate descriptive techniques, which revealed the temporal evolution of scientific production, the most productive and influential journals in the field of open data, and the most frequently used authors' keywords over time.

Second, the data were examined using network and factorial data analysis techniques to discover the conceptual structure underlying the study of open data—that is, the main scientific research themes and trends. Three techniques were employed to detect the conceptual structure through the identification of the relations among concepts or words in the set of publications selected, also adopting a longitudinal framework: in particular, a keywords co-occurrence network, a multiple correspondence analysis and a thematic plot were produced. The authors' keywords, understood as a representative element which provides a definition and synthesis of the content of publications, was used as the unit of analysis in applying these analyses. This choice is in line with the majority of works concerning bibliometric analysis (e.g., Cuccurullo et al., 2016; Pesta et al., 2018; etc.).

The authors' keyword co-occurrence analysis is based on the co-words network in which the nodes are represented by words, and the links define the relationships among them according to their co-occurrences in a set of documents. Two keywords are connected if they are jointly present in two or more documents, and the edges weigh the strength of the associated relationship in terms of number of co-occurrences. Detecting the association between words in the bibliometrics data through their common presence in different documents allows for the identification of the topics covered by a research field as well as the most important and recent issues, that is, the so-called research front (Callon et al., 1983). A clustering analysis based on the Louvain algorithm revealed associations that enabled the identification of different semantic groups that better defined the topics in the scientific field.

A different but coherent perspective emerges through the multiple correspondence analysis (MCA), a multi-dimensional technique used in textual analysis to synthesize the information contained in a data matrix through the identification of a small number of dimensions or factors expressing the relations between variables (Greenacre, 1984). It enabled the exploration of some latent traits of the co-occurrence data structure by highlighting some dimensions of interest and identifying research subfields. By applying this technique and visualizing the results through the factorial plot, it was possible to detect clusters of words whose proximity indicates shared content: keywords close to each other indicate that a high proportion of publications use them together, whereas those that are distant indicate that only a few articles use these words together.

Lastly, a thematic plot was implemented to better define the conceptual structure underlying the studies of open data and, in particular, the overall temporal evolution of the conceptual subthemes. This is a bidimensional plot proposed by Cobo et al. (2011) and based on the network statistics calculated on clusters obtained from the authors' keyword co-occurrence network. The graph was built according to two values: Callon's centrality (*x*-axis) and Callon's density (*y*-axis) measures (Callon et al., 1991) of the nodes. This allowed for the classification of clusters using the Louvain clustering algorithm in terms of the level of development (indicated by the density of connections within clusters) and importance (indicated by the centrality measure of the cluster). In particular, according to the position of the clusters on the graph, it was possible to visually identify the different types of subthemes depending on the quadrant in which they are placed (Cobo et al., 2011). As will be shown in the next section, the so-called *motor* themes are characterized by strong density and centrality values and are positioned on the upper-right quadrant and represent well-developed topics that play a central role in the structure of a research field. By contrast, general and *basic* topics are placed on the lower-right quadrant and present low density and high centrality values. In the upper-left quadrant, there are the *niche* themes, that is, highly developed and isolated themes with high density and low centrality (well-developed internal ties but unimportant external ties). In the lower-left quadrant, there are *emerging* or *declining* themes characterized by low density and centrality.

Lastly, evolution thematic analysis was carried out for three specific time spans (2013–2016, 2017–2019, and 2020–2022) in order to determine the emergence, changes, or decline in the main research trends on open data. Each period was analyzed according to the most recurring author keywords, and the word co-occurrences allowed the identification of the connections between time spans.

## 3. The conceptual structure of the studies on open data

The first results provide descriptive information on the publications collected. The frequency distribution of the scientific publications released in the years 2013–2022 reveals an upward trend, with an annual growth rate of 30.1%. In particular, 2021 represented the year with the highest number of publications. The analysis of the most relevant sources indicates the multidisciplinary nature of studies regarding the topic of open data—within the vast field of human and social sciences—which attracts the interest of journals from different disciplinary fields.

More generally, the results show that the main disciplinary fields—using the Web of Science classification—with the highest number of publications on open data on a theoretical and empirical level are *Social Sciences Interdisciplinary, Public Administration, Environmental Studies, Urban studies*, and *Psychology Multidisciplinary*. These are followed by the strand of *Educational Research, Communication, Management, Political Science*, and *Law*. To a lesser extent, we find *Cultural Studies, Criminology Penology, Ethics, Sociology, Demography, Anthropology*, and *Social Work*.

In particular, journals in the sectors of urban planning, environmental research, and sustainability (“Environment and Planning B-Urban Analytics and City Science,” “Cities,” “Sustainability,” etc.) and in the sectors of psychology (e.g., “Psychological Science,” “Advances in Methods and Practices in Psychological Science,” etc.) are very productive, especially in terms of open data applications. Journals on big data and society (e.g., “Big Data and Society”) also include works on open data. Other publications are in journals that are strictly specialized in the topic of open data (e.g., “Open Data and the Knowledge Society”) and in the wider sector of digital humanities (e.g. “Digital Humanities Quarterly” and “Digital Scholarship in the Humanities”). With regard to scientific production by country, the results indicate that the United States has the highest number of publications, followed by the United Kingdom, Germany, Netherlands, China, and Italy, which over the years 2013–2022 show a rising trend in the number of publications concerning open data and its applications.

A central part of this research concerns the understanding of the trending topics, the different themes and perspectives related to open data, achieved through the analysis of authors' keywords.

Examining the frequency distribution of authors' keywords over the time span considered in this work reveals that the most frequently used keywords on the main issues are strictly related to the characteristics of open data and its interconnection with other types of digital data, such as *transparency, linked open data, big data*, and *e-government*. Other commonly-used keywords concern works on the policy-making processes and the most recent pandemic emergency, such as *COVID-19* and *decision making*. Other widely used keywords refer to specific areas in which open data are used, such as *smart city, education, social*, and *learning*. The recurrence of these themes is highlighted below, also through the results obtained through the various analyses carried out, which points to the study of open data in its role of rethinking, monitoring, and taking action in different contexts of public services and in the improvement in the quality of life, such health care, democratic participation, education, and smart cities.

**Figure 1:**
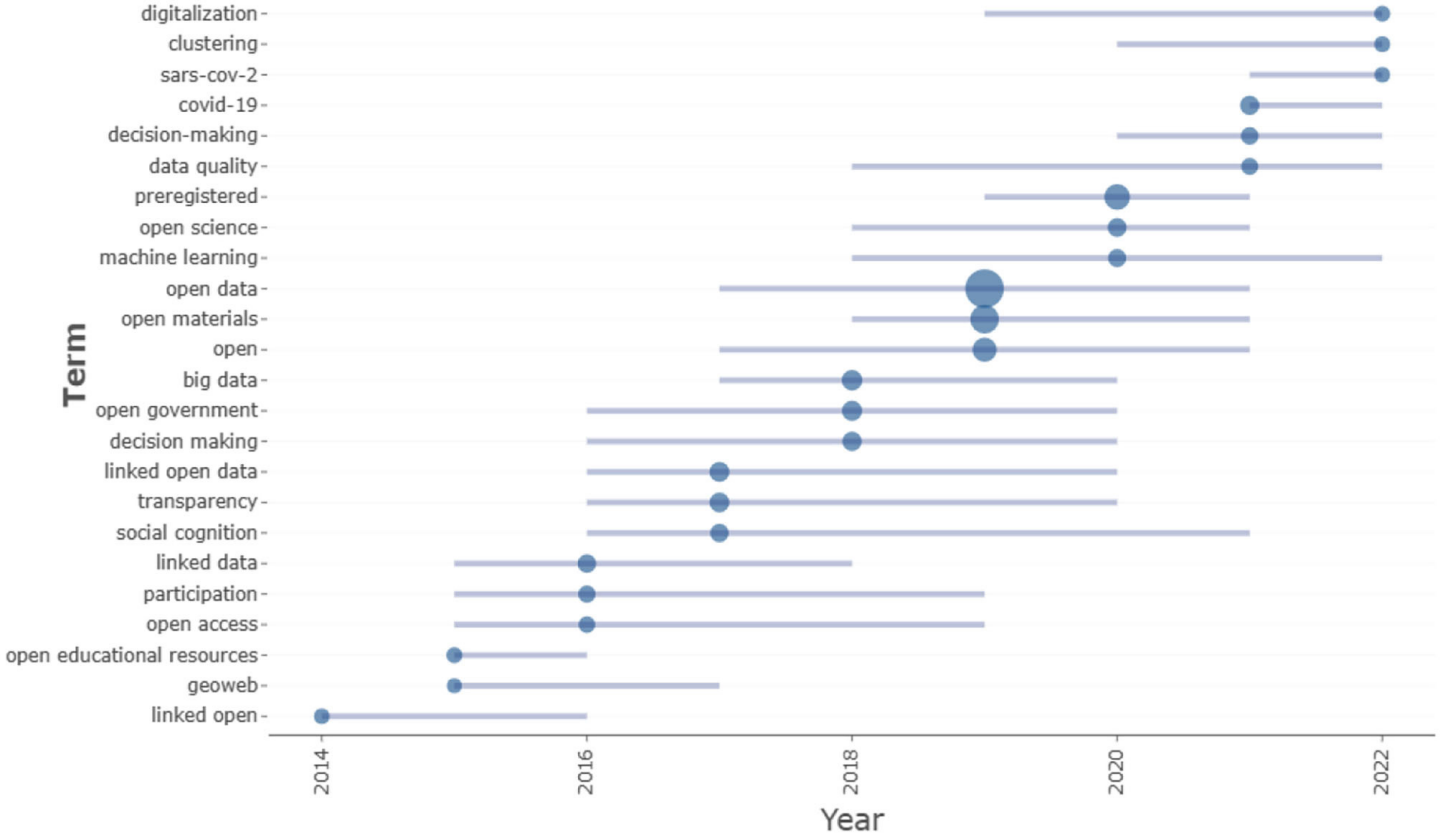
Trend topics.

These results are better outlined by analyzing the period of the greatest use of authors' keywords (Figure 1). Exploring the distribution of higher-frequency keywords over time, indicated by the size of the circles and by the length of the lines in the Trend topic plot, two related but different temporal configurations emerge. In the first period starting from 2014, the topic of open data is mainly addressed through the study of some dominant as well as basic themes such as *participation, open access, transparency*, and *linked open data*. During the second period starting from 2017, the most commonly used keywords in the publications collected concern multiple aspects related to the characteristics of the data and processing and analysis of open data, such as *machine learning, data quality*, and *big data*. This result, which also emerges in the analysis below, is interesting, considering that publications belonging to the “hard sciences” were excluded in this research. It suggests how the themes on the processes of quantification and measurement are converging in a social and human sciences-informed perspective of data culture (Micheli et al., 2020).

Additionally, one specific characterization concerns the years 2021 and 2022, in which open data topics are related to decision making and the COVID-19 phenomenon. This result, which also emerges in other analyses, underlines how the sharing of open datasets took on an important role in understanding the progression of the virus and in improving the response of the different administrations to the pandemic (Desvars-Larrive et al., 2020).

After identifying the most frequently used keywords, the conceptual structure underlying the study of open data was explored using different data analysis techniques, such as authors' keyword co-occurrence network, the MCA, and the thematic plot.

**Figure 2:**
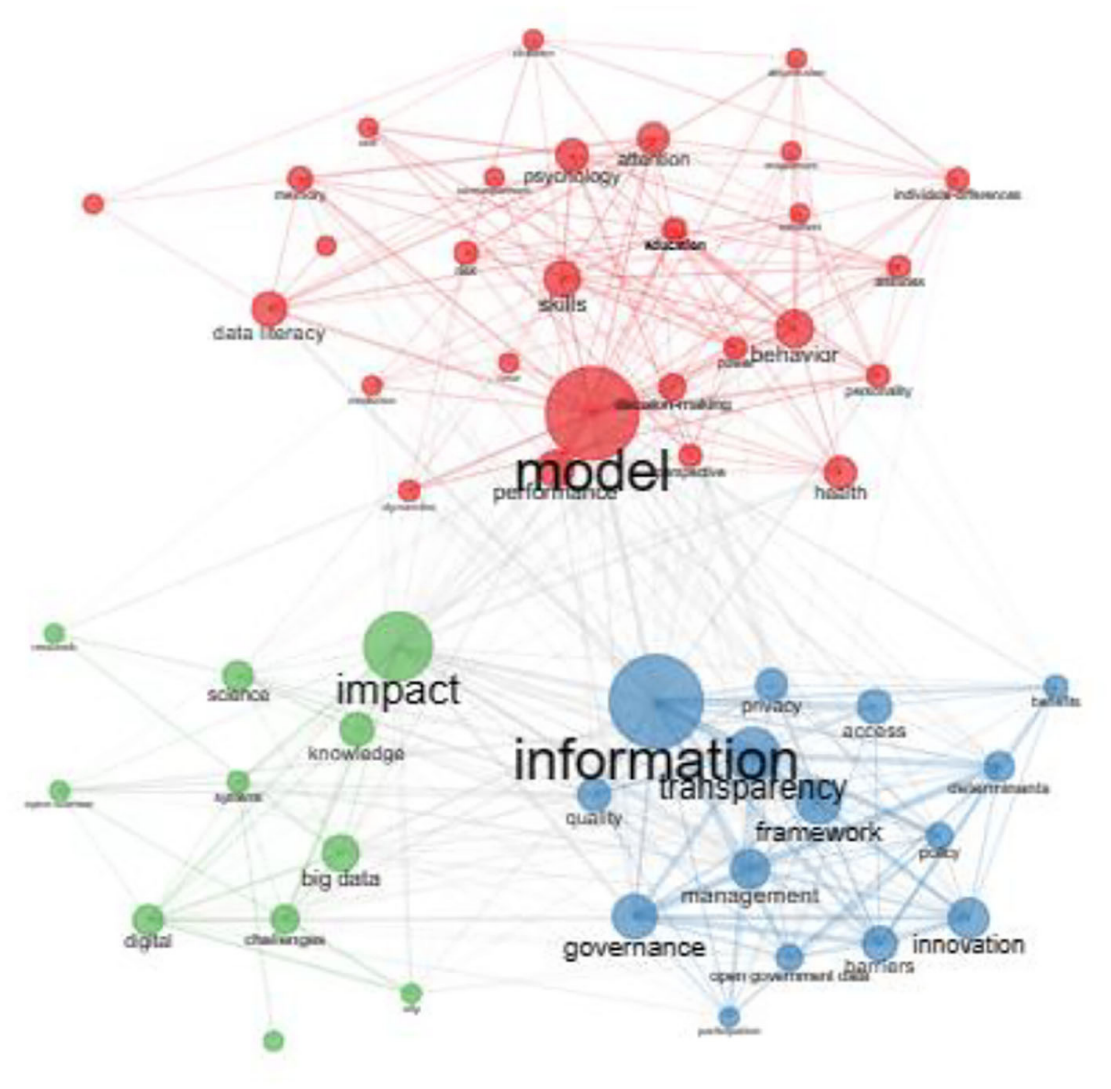
Authors' keyword co-occurrence network.

The authors' keyword co-occurrence network allowed us to discover the connections between keywords, detecting some specific features of studies on open data. The results in Figure 2 show a network of three clusters representing different subfields in studies of open data.

The first cluster of keywords (shown in blue) revolves around the processes of open data management and governance. In fact, this group places the term “*information*” almost at the center of the network around which the main keywords are: *access, policy, transparency, management, governance, privacy, benefits, barriers, determinants*, and *framework*. The publications of this group regard the regulatory and also the political settings that relate to the complex definition and management of policy, roles and conditions, underpinning open data, as well as the plurality of critical issues related to the transparency, quality and traceability of internal processes, characterizing the debate on open data.

The second cluster of keywords (shown in green) indicates how the publications analyzed seem to deal with the relationship between data and knowledge processes and its new frontiers, converging toward the direction of open science approaches up until the interconnections with the spread of big data. The main keywords characterizing this cluster are *knowledge, science, open science, big data, digital, impact, systems*, and *challenges*. The proliferation of open data and also the increasing process of datafication expressed by the development of big data contribute to the ongoing transformation of knowledge (Baack, 2015). However, the vision underpinning open data is mainly based on the sharing and use of structured data linked to collaborative forms of government, unlike big data whose construction and dissemination processes are often opaque (Baack, 2015).

In other words, these two clusters suggest that the scientific studies on open data focused on the one hand on the processes of data governance, in terms of data access and management, and on the other hand on the interconnections between digital development—in its various dimensions—data production (open data and big data) and knowledge. In this perspective, the concepts representing these clusters of keywords pose central questions about the growing process of datafication that characterizes the digital society: how information is organized and managed and the impact on the nature of knowledge (Lievrouw, 2011, p. 26; Baack, 2015).

The third cluster (shown in red) brings together publications dealing with behavioral *models*. The main keywords of this cluster, revolving around *model* keywords, are *attitudes, behavior, personality, self*, *emotions, perception, literacy, skills*, and *education*. The keywords in this cluster refer to studies that seem to be interested both in educational aspects related to the development of data understanding and literacy skills, and in aspects more inherent to the psychological and social dimension, which may represent possible contexts for the application of open data.

Next, an MCA was conducted to focus specifically on the conceptual structure underlying the study of open data. This multidimensional technique detected interdependence among a set of lexical data and identified new latent traits of the co-occurrence data structure. In particular, the interconnections between the themes of the clusters previously obtained within the authors' keyword co-occurrence network are analyzed in greater depth.

**Figure 3:**
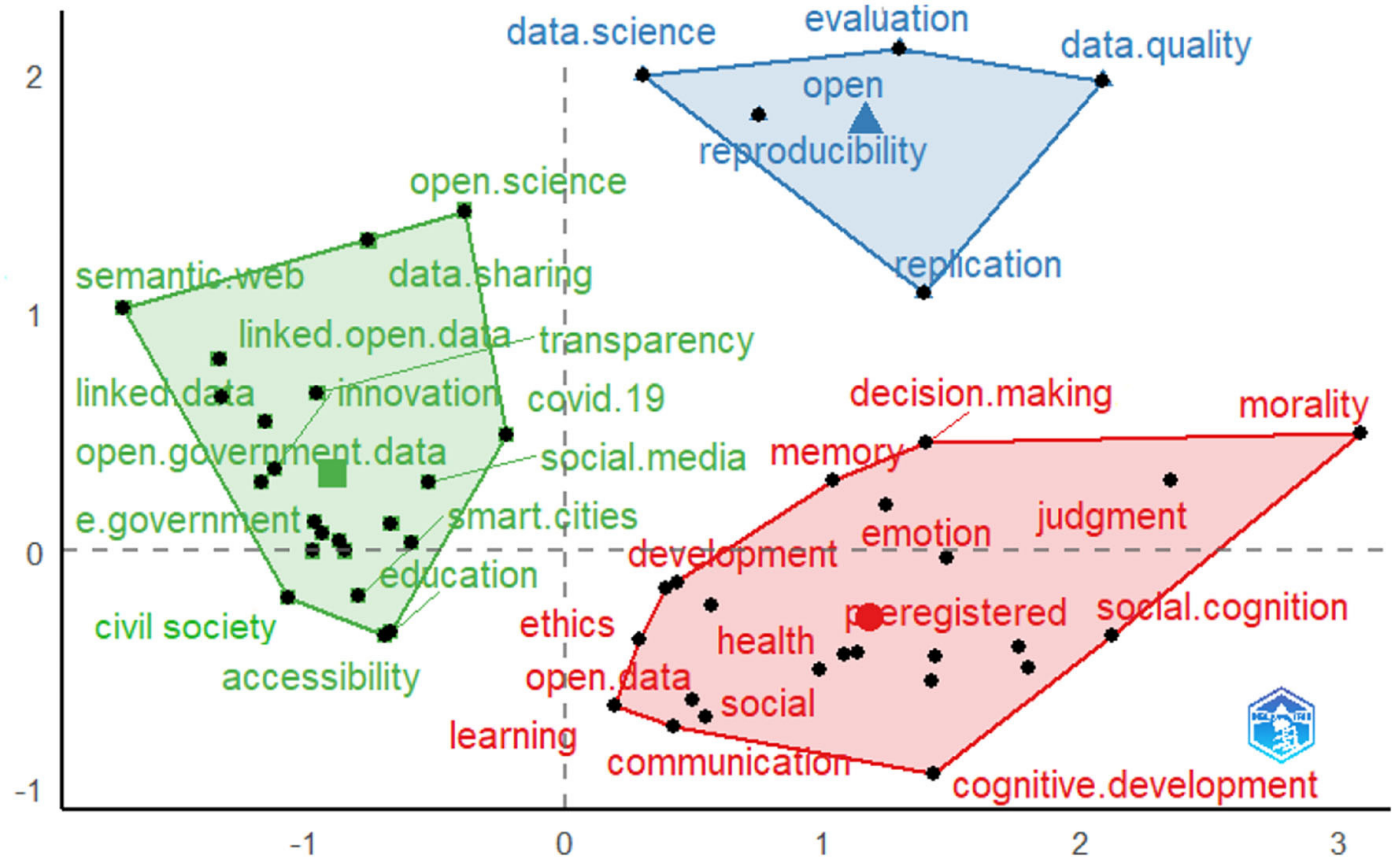
Multiple correspondence analysis.

In the factorial plot shown in Figure 3, the right-hand side of the x-axis is characterized by keywords (marked in red) that relate more to the application contexts of open data, such as *health, learning*, and *communication*, and to the more psychological aspects, such as *emotions* and *cognition*. Moreover, in this quadrant, it is possible to find a further characterization of the third cluster described in the previous analysis concerning studies on the behavioral models, as it is also made up of lemmas related to the dimension of *ethics*, which raises questions, for instance, when striking a balance between the openness of public information assets and the protection of personal data. The left side of the x-axis (marked in green) brings together keywords mainly concerning the processes of information management related to the concepts of: *data sharing, transparency, accessibility, e-government*; other keywords of this cluster suggest studies on the interconnections that *data sharing* processes have with *civil society*, urban development (*smart cities*) and the management of the recent pandemic (*COVID-19*). Lastly, the quadrant above the others identifies a group of keywords (marked in blue) related more closely to dealing with the basic prerequisites and standards that characterize the scientific method associated with the study and application of open data in terms of *data quality, data science, replicability, reproducibility*, and *evaluation*, etc. For example, the scientific debate in the field of social sciences is dealing with the question of *data quality* and the *reproducibility* of digital data—and also open data—produced in the digital platforms, understood as a social artifact with certain logics, aims and constraints, often not determined and not accessible by the researcher (Capogna, 2022).

**Figure 4:**
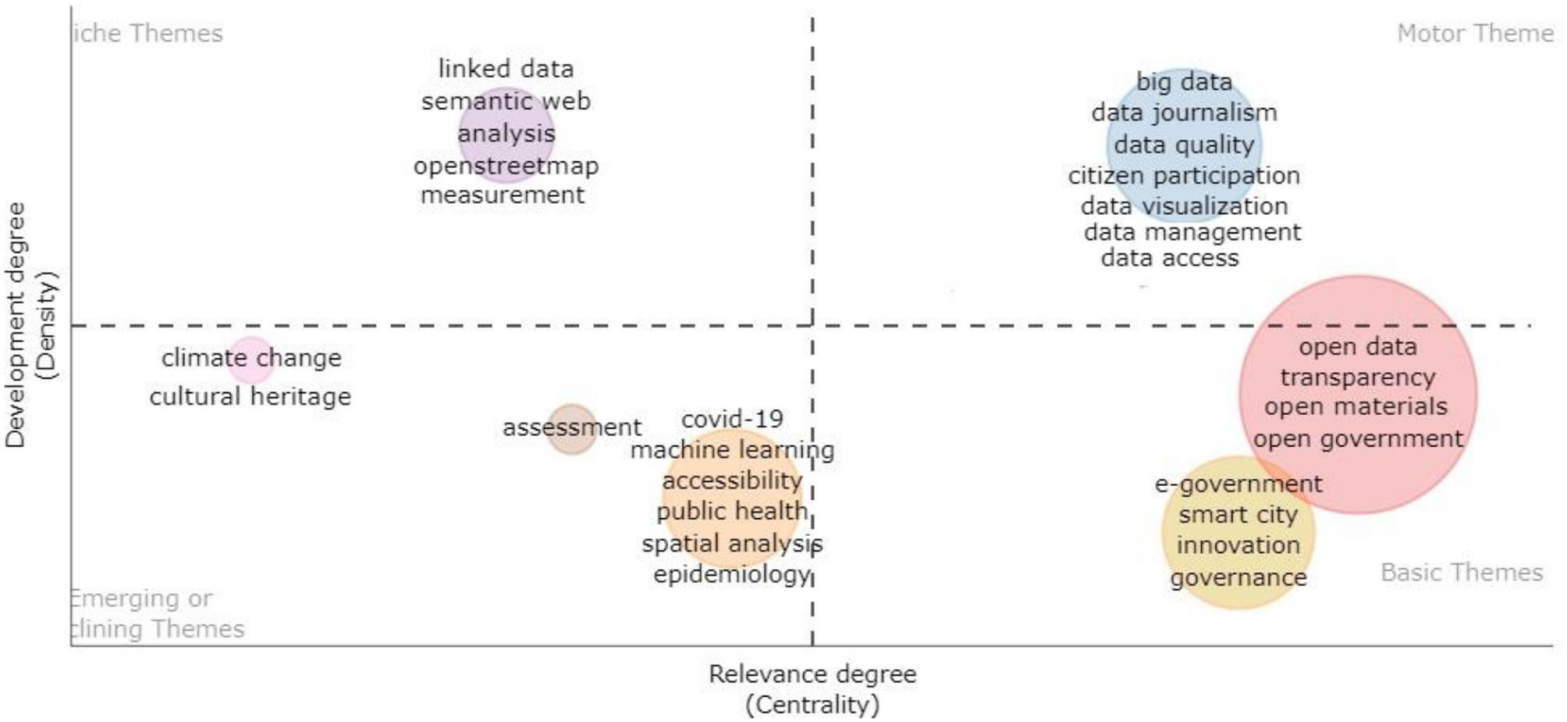
Thematic plot.

To better understand the conceptual structure underlying the study of open data over time and analyse the evolution of the topics in the 10-year period, a thematic plot was created (Figure 4). This map, obtained using the R-Bibliometrix tool, presents the current state of the sector and the potential for future research development in specific thematic areas. More specifically, it represents clusters of keywords according to the values of centrality (which detects the importance of the theme, as it measures the degree of correlation among different topics) and the density (which indicates the theme's development, as it quantifies the cohesiveness among the nodes) (Esfahani et al., 2019; Agbo et al., 2021). Based on the quadrant in which they are placed it was possible to identify four themes: *motor, basic, emerging* or *disused* and *niche*.

The upper-right quadrant is characterized by *motor* themes, i.e., themes that are highly developed, mature, and established in scientific research and are leading topics within the discipline. They concern the different aspects and processes that revolve around the specific concept of data, such as *quality, visualization, management*, and *access*. Motor themes also include *big data*, which in some ways and as shown in previous analyses intersect with open data studies. These results suggest that these topics, while traditionally closer to the “hard” sciences, are also being addressed by the humanities and social sciences, supporting a more human-centered perspective that, for example, leads to problematising measurement conventions and the complex issues (e.g., data quality and data access) underlying the construction of detection systems.

The lower-right quadrant represents the *basic* themes of scientific production, that is, areas capable of defining the research field, which in this perspective play a significant role in the development of the field. They concern the core concepts of the open data approach and touch on issues related to transparency and open government processes (*transparency, e-government*, and *open-government*), with others related to the impact of open data on innovation pathways and urban development (*innovation, smart city*) (Berrone et al., 2016; Davies and Perini, 2016; Neves et al., 2020).

The bottom-left quadrant displays the so-called *emerging* or *disused* themes, characterized by low values of centrality and density. These issues can be described as “peripheral,” as they are new to the research field or, instead, have ceased to be integrated in the discipline. The majority of these mainly concern the different contexts of application of open data and are linked to the most recent historical period characterized by the COVID-19 pandemic, as indicated by the following keywords: *public health, epidemiology*, and *COVID-19*. Moreover, some sectors seem to have an emerging and increasing use of open data, such as *climate change, cultural heritage*, and *assessment*.

Other topics placed in this quadrant also deal with some aspects related to data processing, such as *machine learning* and *spatial analysis*. At the same time, the themes connected to the open data analysis, such as *measurement, analysis*, are placed in the top-left quadrant. According to the high value of the density and low value of the centrality, they represent *niche* topics, that is, a highly developed research field with strong internal relationships but isolated and marginal (i.e., with weak external relationships). Notably, these aspects concern the analysis and processing of open data and appear to be niche themes, perhaps because the bibliometric analysis focused on a set of publications extracted from the social and human sciences sectors.

**Figure 5:**
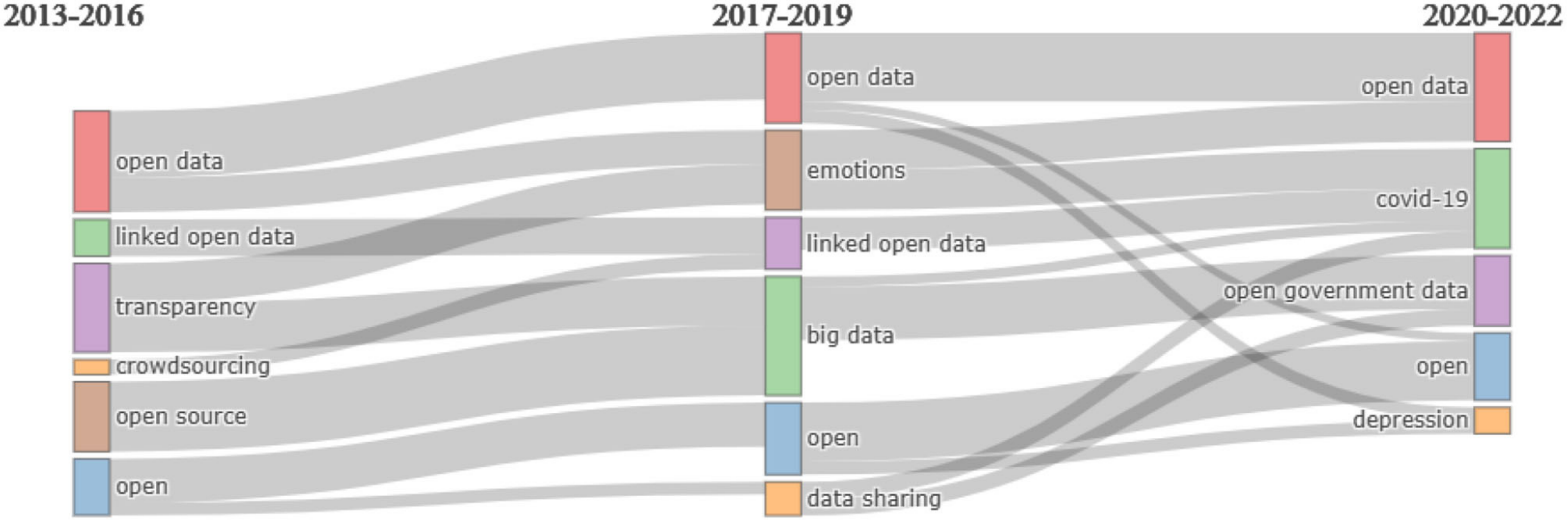
Thematic evolution.

Finally, a thematic evolution analysis was conducted for three-time spans (2013–2016, 2017–2019, and 2020–2022) in the order to trace the temporal evolution—in terms of emergence, changes, or decline—of the main research themes on open data (Figure 5). Each period is analyzed with respect to the most frequently recurring author keywords, which become representative of the reference period; in addition, in this analysis the word co-occurrences reveal connections between time spans. The main results showed the persistence of *open* and *open data* keywords that characterize all periods, i.e., the continuity of the debate over time on these concepts that still raise a definitional concern. As Mayernik (2017) points out, the concept of “open data” itself continues to face several central definitional issues, especially with respect to the concept of “open” (Levin et al., 2016).

Moreover, it is interesting to observe the presence of some topics in each period, such as *transparency* in the first timeframe (2013–2016), *big data* and *emotion* in the second (2017–2019) and *COVID-19* and *open government* in the third (2020–2022). In particular, the theme of *transparency* and *open source* that characterizes the 2013–2016 years flows into the theme of *big data* during 2017–2019, which in turn converges into the theme of *open government* data in 2020–2022. Another interesting path, again inherent to the theme of *transparency*, is its link with the theme of *emotions* during 2017–2019, which in turn converges with the theme of *COVID-19* in 2020–2022.

## 4. Discussion and concluding remarks

The aim of this work was to explore the main research patterns and trends in the scientific literature of the social sciences and humanities produced during the years 2013–2022 on the topic of open data through bibliometric analysis. In addition to the upward trend in the number of publications during the period considered, the various analyses conducted showed that the literature on open data covers different topic areas that converge mainly in three main aspects: topics that characterize the disciplinary and foundational domain of the open data approach, such as *data sharing, transparency, open-government*, and *accessibility*. In part, these concepts represent the basic themes underpinning scientific studies on open data.

This is followed by publications on more specific topics related to methodological issues and procedures related to open data (*quality, data science*, and *replicability, reproducibility*, and *evaluation*, as well as *knowledge, science, big data, cities, impact*, and *challenges*), as well as those influencing new forms of knowledge. In particular, these studies dealt with how digital data, such as open data and big data, can redefine the key questions that drive scientific knowledge, exploring measurement conventions (Salais, 2016), the processes involved in building automated detection systems, and the issues of internal consistency of the theoretical-methodological framework (Capogna, 2022).

The third strand of studies concerns the areas of application of open data around both social and psychological behavior *models* and captures more recent phenomena in contemporary society, ranging from *climate change, smart cities*, and *cultural heritage* to the recent health emergency of the *COVID-19* pandemic.

One significant aspect that seems to be a common thread in open data studies and that mainly characterizes the scientific production of the first years analyzed, serving as a basic theme, is *transparency*. Considered as “the notion that information about an individual or organisation's actions can be seen from the outside” (Mayernik, 2017, p. 2), the topic of transparency in open data studies is explored in its different forms: the relationship between *transparency* and *open governance*, a central aspect on which the relationship between institutions and citizens can be based. Other studies address the connection between *transparency* and *data* in the narrow sense, in its interconnections with traditional and emerging forms of scientific knowledge and the resulting ethical and methodological implications. Thus, in the various analyses, and especially in the thematic evolution analysis, it is no coincidence that in the publications on open data analyzed, the topic of *transparency* flows into the 2020–2022 studies of *big data*.^1^ This relationship brings up a number of issues, such as the traceability of internal processes in the phases of data construction and analysis, which are increasingly the subject of scientific debate.

Overall, the results show a growing interest of the social sciences and humanities in the various components and aspects that characterize the culture of data in its different forms (e.g., *quality, data sharing, reproducibility*, and *big data*), in the epistemological and methodological implications and the social, political and educational interconnections. This is an interesting result considering that in the past various scholars (e.g., Savage and Burrows, 2007) had shown some concern about the process of increasing digitisation, which in this sense could have diminished the role of the human and social sciences as a public form of knowledge, shifting the analysis of social phenomena to hard science experts (mathematicians, computer scientists, physicists, etc.), capable of handling the multiplicity of data inhabiting digital contexts (Lombi, 2015, p.215).

However, the different degrees of interest found in the disciplinary fields analyzed regarding the topic of open data is worth mentioning. Also taking into account the univariate descriptive analyses, open data is most frequently explored through the study perspectives connected to *Public Administration, Urban studies*, and *Multidisciplinary Psychology*; but a high level of interest is also found for the fields of *Education, Communication, Management*, and *Political Science*. On the other hand, the reflection on the interconnections between open data and the more strictly cultural-ethical and sociological components are beginning to emerge: the fields belonging to *Cultural Studies, Sociology, Criminology, Ethics, Anthropology*, and *Social Work* have fewer publications on this topic, although the number rose over the years analyzed. In addition, the results on scientific production by country also suggest how research interest is closely linked to the socio-economic and cultural contexts in which it develops and how it is distributed differently in the various countries: there is a greater response from scholars from institutions in the USA and Europe (especially from the United Kingdom, Germany, Netherlands, and Italy) and China. To a residual extent by scholars from institutions in economically and socially disadvantaged countries such as various eastern countries (e.g., Kazakhstan, Mongolia) and African countries (e.g., Ghana, Sudan, Algeria).

The main findings of the research presented in this article also show various conceptualisations of the meanings and approaches on the topic of open data adopted by different publications and in different time periods. The pluralism of perspectives underlying the study of open data is not ascribed to a unified paradigm but is related to the need to cross-promote an interdisciplinary interpretation, a critical discussion around the processes of creating, using, sharing, and interpreting open data, that is, around data culture. Hence, the results obtained here indicate how the pervasiveness of data requires a significant ability to know and interpret their language and thus of the complex set of theoretical, logical, and operational steps that link abstract concepts to their empirical correlates through a process of signification. In line with the findings of other studies, the aptitude to combine different languages and points of view seems to be necessary to understand the information capacity of this type of data, and consequently to be able to support political and governance processes and choices of collective interest at all levels (Capogna, 2022).

Studying open data as a heterogeneous topic makes it possible to recognize its peculiarities in terms of benefits but also in terms of criticalities, barriers and negative impacts. With respect to this last point, only in part do studies seem to address the negative consequences triggered by open data. For instance, the complex issue of *data quality*—which emerged in the analyses conducted here—and the lack of evaluation procedures are connected to the chain of decision-making processes—institutional etc.—which risk also being based on poor information quality (Zuiderwijk and Janssen, 2014). Another issue concerns the educational sphere and in particular the level of data literacy on the part of the different actors (citizens and other stakeholders), which differs according to socio-economic-cultural contexts and can therefore generate forms of misinterpretation. Zuiderwijk and Janssen (2014) in this regard illustrated the dark sides that accompany the scientific reflection on open data: e.g. privacy violations especially due to combining data with other sources; the variety of interests that do not only concern citizens but different types of stakeholders. Concerning this point, the vision of open data can be linked to a neo-liberal logic in which private stakeholders support the commercialization and marketisation of public services for financial gain (Bates, 2012; Martin, 2014). According to the study of Zuiderwijk and Janssen (2014), an important issue concerns the informational value of the data to be made open, as at times there is a waste of resources in publishing data that is of little significance. This issue is also closely related to the risks of information overload that can sometimes result in increased confusion, and less trust and understanding (Janssen et al., 2012).

These aspects therefore highlight the need to reflect on the deeper meaning of data, contemplating the different uses, contexts and ways in which they can be used to address certain challenges of contemporary society and in this sense be generators of public value. The results obtained—which show a growing interest of the social sciences and humanities—seem to be moving in this direction, oriented toward overcoming the “data driven” approach—often characterizing the vision of data sharing—and understanding the social significance and relevance underlying the combination of data and technologies.

## Data availability statement

The data for this study was collected from the Web of Science database and is available upon request.

## Author contributions

The author confirms being the sole contributor of this work and has approved it for publication.

## Footnotes

^1^Big data, understood as a complex assemblage of human and non-human components (Aragona et al., 2018), are somehow connected to open data, representing, according to various studies, a broader landscape in which open data is also located.
